# The Niche Factor Syndecan-1 Regulates the Maintenance and Proliferation of Neural Progenitor Cells during Mammalian Cortical Development

**DOI:** 10.1371/journal.pone.0042883

**Published:** 2012-08-24

**Authors:** Qingjie Wang, Landi Yang, Caroline Alexander, Sally Temple

**Affiliations:** 1 Neural Stem Cell Institute, Rensselaer, New York, United States of America; 2 Center for Neuropharmacology and Neuroscience, Albany Medical College, Albany, New York, United States of America; 3 McArdle Lab for Cancer Research, University of Wisconsin-Madison, Madison, Wisconsin, United States of America; Instituto de Medicina Molecular, Portugal

## Abstract

Neural progenitor cells (NPCs) divide and differentiate in a precisely regulated manner over time to achieve the remarkable expansion and assembly of the layered mammalian cerebral cortex. Both intrinsic signaling pathways and environmental factors control the behavior of NPCs during cortical development. Heparan sulphate proteoglycans (HSPG) are critical environmental regulators that help modulate and integrate environmental cues and downstream intracellular signals. Syndecan-1 (Sdc1), a major transmembrane HSPG, is highly enriched in the early neural germinal zone, but its function in modulating NPC behavior and cortical development has not been explored. In this study we investigate the expression pattern and function of Sdc1 in the developing mouse cerebral cortex. We found that Sdc1 is highly expressed by cortical NPCs. Knockdown of Sdc1 in vivo by in utero electroporation reduces NPC proliferation and causes their premature differentiation, corroborated in isolated cells in vitro. We found that Sdc1 knockdown leads to reduced levels of β-catenin, indicating reduced canonical Wnt signaling. Consistent with this, GSK3β inhibition helps rescue the Sdc1 knockdown phenotype, partially restoring NPC number and proliferation. Moreover, exogenous Wnt protein promotes cortical NPC proliferation, but this is prevented by Sdc1 knockdown. Thus, Sdc1 in the germinal niche is a key HSPG regulating the maintenance and proliferation of NPCs during cortical neurogenesis, in part by modulating the ability of NPCs to respond to Wnt ligands.

## Introduction

Emerging from the dorsal telencephalic neuroepithelium, the mammalian cerebral cortex develops into a highly organized and complex structure. NPCs, which include neural stem cells (NSCs) that self-renew and restricted progenitor cells that amplify the lineage, are the founder cell population for this remarkable process [1], [2] .There are two major types of NPC during cortical neurogenesis. Radial glial cells (RGCs), which are also known as apical progenitor cells as they have their soma immediately next to the ventricle in the ventricular zone (VZ), are the principal progenitor cells for cortical pyramidal neurons [3], [4]. RGCs initially undergo symmetric divisions that expand the progenitor population, then switch to asymmetric divisions concomitant with the initiation of neurogenesis [3], [5], [6]. Intermediate progenitor cells (IPCs), also termed basal progenitor cells, originate from RGCs, typically by an asymmetric cell division that gives an RGC and an IPC that then delaminates into the secondary germinal layer, the subventricular zone (SVZ) [7], [8], [9]. IPCs act as transit amplifying cells with restricted potency, dividing once or twice to generate 2–4 neurons [7]. The new born neurons migrate along RGC processes to the cortical plate and there build the six layers of neocortex in an “inside-out” manner, with early-born cells forming the deep layers and later-born cells forming the superficial layers [10]. Proper construction of the cerebral cortex is achieved through precisely balanced self-renewal, proliferation and differentiation of NPCs.

Several exogenous growth factors and cytokines have been shown to regulate the balance between NPC proliferation and differentiation during cortical neurogenesis. Among them, fibroblast growth factors (FGFs), epidermal growth factor (EGF), Wnt and vascular endothelial growth factor (VEGF) positively regulate NPC proliferation [11], [12], [13], [14] while other exogenous factors such as bone morphogenetic proteins (BMPs), ciliary neurotrophic factor (CNTF) and cardiotrophin-1 promote differentiation into first neurons and later glia [15], [16].

The distribution, stability and activity of many environmental factors rely on proteoglycans, which are present in the extracellular matrix and associated with cell membranes [17]. Syndecans are the major family of transmembrane HSPGs. Four members have been identified in mammals: syndecan-1, syndecan-2/fibroglycan, syndecan-3/N-syndecan, and syndecan-4/amphiglycan [18], [19]. Each syndecan has a specific expression pattern and therefore likely a unique function in the brain [20]. Syndecan-2 locates at synapses and is critical for dendritic spine maturation in hippocampal neurons [21]. N-syndecan is abundantly expressed in neuronal axons and regulates neuronal migration [22], [23]. Syndecan-4 is expressed in astrocytes and regulates adhesion [24], syndecan-4 is also expressed by astroglia as the angiogenin receptor and mediates specific uptake of angiogenin [25]. Syndedcan-1 (Sdc1) is highly enriched at early neural germinal zones prior to neurogenesis [26] and its expression level decreases as cortical neurogenesis proceeds [27] and there is no detectable signal for Sdc1 in adult forebrain [20], suggesting that Sdc1 plays a unique role in cortical neurogenesis.

In this study we present evidence for the function of Sdc1 during mammalian cerebral cortical neurogenesis. By immunohistochemistry we show that Sdc1 is specifically enriched in the neural germinal zone of developing cortex on both of the major progenitor classes, the RGCs and IPCs. Knockdown studies show that Sdc1 is important for the maintenance and proliferation of NPCs, both in vitro and in vivo. Furthermore, we found that Sdc1 modulates the ability of NPCs to respond to Wnt ligand. Together these data demonstrate that Sdc1 is a key germinal niche environmental factor that regulates the behavior of NPCs during mammalian cortical neurogenesis.

## Materials and Methods

### Ethics Statement

This study was carried out in accordance with the care and use animal protocols approved by the University at Albany (UA) Institutional Animal Care and Use Committee (#10-010). The UA facility has filed a written assurance with the Public Health Service and we are committed to comply with the Guide for the Care and Use of Laboratory Animals and the provisions of the Animal Welfare Acts.

### Plasmid and Lentivirus Preparation

shRNA plasmids were generated by inserting the hairpin oligonucleotides (Scrambled control: TTCTCCGAACGTGTCACGT; Sdc1 ORF: CCACACCTGTCGTCCACTC; Sdc1 UTR: TGTCATTGCCGGAGGCCTA) into the FUGW-H1 lentiviral construct as previously described [28]. To package the lentivirus, the constructs were co-transfected with pCMV-VSVG and pCMV-dvpr into 293FT cells. Supernatant was harvested 2 and 3 days later and further concentrated by ultra-centrifugation. Lentiviruses were used at 10 MOI for cell transduction.

### Cell Culture

For adherent cultures, embryonic cortices from timed pregnant Swiss Webster mice (Taconic Farms) were dissected and dissociated into single cells, and cultured as described previously [29] in serum free DMEM medium with 10 ng/ml FGF2 (Invitrogen) on PLL (Sigma) coated 48 well-plates. For clonal analysis, 3,000–5,000 cells were plated per well. Chir 99021 (Stemgent) was used to block GSK3β activity. Wnt3a conditioned medium was harvested and concentrated from L cells stably expressing mouse Wnt3a (ATCC number: CRL-2647™). For neurosphere cultures, single dissociated cortical cells were plated in serum-free DMEM medium with 20 ng/ml FG2 and EGF (Invitrogen) in ultra-low binding 6 well plates (Corning). Cells were plated at 6,000 cells/well and neurosphere numbers were counted seven days later.

### In Utero Electroporation

Pregnant mice (Swiss Webster, Taconic Farms) were anesthetized and the uterus exposed. Plasmid was injected into the embryonic forebrain lateral ventricle (∼1 ul per injection, 2 ug/ul concentration) and electroporation was performed using an electroporator (BTX) at 35 volts for 5 pulses, each pulse lasting 50 ms, 900 ms apart. The uterus was gently repositioned, and the abdominal wall and skin closed with a needled suture. Embryos were harvested two days later and the brains dissected and fixed with 4% paraformaldehyde overnight, followed by 30% sucrose/PBS treatment overnight, then embedded in OCT, frozen on dry ice and cryostat sectioned into 16–30 um coronal sections. For electroporation analysis, two sections per embryo were used to calculate the average number of GFP+ cells in each condition located between the VZ and CP (Width of the window area used to count the GFP+ cells: 250 um–300 um), matching electroporation location between experimental and control groups. The efficiency of electroporation was similar between experimental and control conditions, and we inspected 3–7 embryos per condition to minimize experimental variability.

### Immunohistochemsitry and Immunocytochemistry

The cryostat sections were incubated in 10% normal goat or donkey serum/0.3% Triton/PBS for 1 hour at room temperature to block the tissue, followed by incubation with primary antibody diluted in blocking buffer at 4°C overnight. After washing three times with PBS, the sections were incubated with appropriate secondary antibodies (Invitrogen) diluted in blocking buffer for 1 hour at room temperature, followed by a 10 minute incubation with Hoechst 33342 (Invitrogen) diluted in PBS. After washing three times with PBS, the sections were mounted with Antifade reagent (Invitrogen) and Apotome (Carl Zeiss) fluorescent microscopy was used for image acquisition.

Citric acid treatment to unmask the antigens Pax6, Tbr2 and Ki67: Sections were incubated in heated citric acid buffer (10 mM sodium citriate, pH 6.0) at 95–97°C for 15 minutes. After cooling to room temperature, the sections were washed three times with PBS, followed by the standard immunohistochemistry protocol, as described.

Cultured cells were fixed and immunostained as previously described [29].

Primary antibodies: Sdc1 (Rat IgG, 1∶100, BD Pharmingen), Sdc1 (Rabbit IgG, 1∶500, gift from Dr. Alan C. Rapraeger), Ki67 (Rabbit IgG, 1∶200, Bethyl Labs), GFP (Chicken polyclonal, 1∶500, Aves Labs), Tbr2 (Rabbit IgG, 1∶200, Abcam), Pax6 (Mouse IgG1, 1∶20, Developmental Hybridoma Bank), Nestin (Mouse IgG1, 1∶4, Developmental Hybridoma Bank), Tuj1 (Mouse IgG2b, 1∶1000, Sigma), Sox2 (Goat IgG, 1∶200, Santa Cruz), β-catenin (Mouse IgG1, 1∶200, BD biosciences), active Caspase-3 (Rabbit IgG, 1∶200, Promega) .

### Real-Time PCR

The mRNA was harvested from cultured neurospheres using the RNeasy Micro Kit (Qiagen), then cDNA was synthesized using the SuperScript® III First-Strand Synthesis System (Invitrogen), followed by real-time PCR analysis using an Applied Biosystems® 7500 Real-Time PCR System.

### Western blots

Protein lysates were harvested from cultured cells or tissue with low salt lysis buffer (50 mM Tris-HCL and 1% NP-40, pH = 8.0) containing HALT protease inhibitor and phosphatase inhibitor (Thermo Scientific). Protein concentration was measured by BCA assay and equal amounts of protein were loaded onto NuPAGE Novex Bis-Tris gels (Invitrogen) and transferred onto PVDF membrane after electrophoresis. Membranes were blocked with 5% milk or BSA in TBST buffer for 1 hour, then incubated with primary antibody in blocking buffer at 4°C overnight. Peroxidase-conjugated secondary antibodies (Jackson Immunoresearch) were applied for 1 hour at room temperature. Supersignal West Pico Chemiluminescent Substrate (Thermo Scientific) was used and the blot signal was visualized using a GE LAS 4000 imaging system. Primary antibodies: Sdc1 (Rabbit IgG, 1∶1000, gift from Dr. Alan C. Rapraeger, University of Wisconsin-Madison), GAPDH (Rabbit IgG, 1∶1000, Cell Signaling Technology), β-catenin (Mouse IgG1, 1∶1000, BD biosciences).

### Statistics

All statistics - t-tests (two-tailed) and one-way-ANOVAs with Bonferroni post hoc tests – were performed with GraphPad Prism software.

## Results

### Expression of Sdc1 in the developing cortex

**Figure 1:**
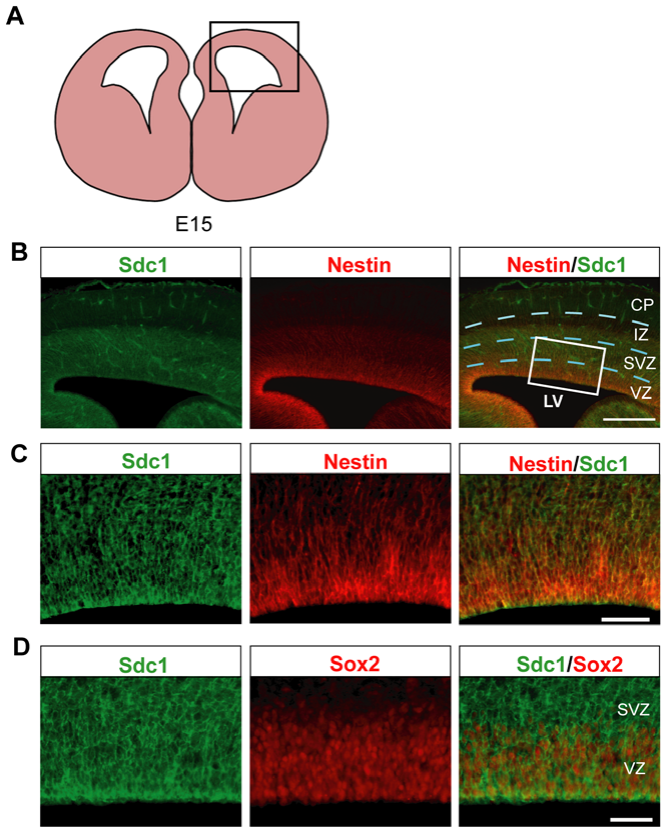
Sdc1 is highly expressed in the germinal zones of the developing mouse cerebral cortex. (A) Schematic to indicate area of analysis. (B) Sdc1 expression in a coronal section of the E15 mouse cortex (area indicated by the box in Fig. 1A). Sdc1 is highly expressed in both the VZ and SVZ (scale bar = 200 um). (C) Higher magnification of the boxed area indicated in Fig. 1B (scale bar = 100 um). (D) Sdc1 expression in the neural germinal zone where Sox2+ RGCs are located (scale bar = 100 um). LV, Lateral ventricle; CP, cortical plate; IZ, intermediate zone; SVZ, subventricular zone; VZ, ventricular zone.

We first examined the spatial expression of Sdc1 in the developing cortex by probing coronal cortical sections. As exemplified by embryonic day 15 (E15) coronal sections (Figure 1A), the Sdc1 signal is highly enriched in both major cortical germinal zones, the VZ and the SVZ (Fig. 1B, 1C). The Sdc1 signal is especially prominent at the apical surface of the VZ where it co-localizes with the Nestin+ endfeet of RGCs, the principal progenitor cells in the VZ (Fig. 1C). Sdc1 is strongly expressed by the RGCs in the VZ, which label with Sox2+, and by the overlying SVZ layer of IPCs (Figure 1D).

### Knocking down Sdc1 results in loss of NPCs and increase in neuronal differentiation in vitro

**Figure 2:**
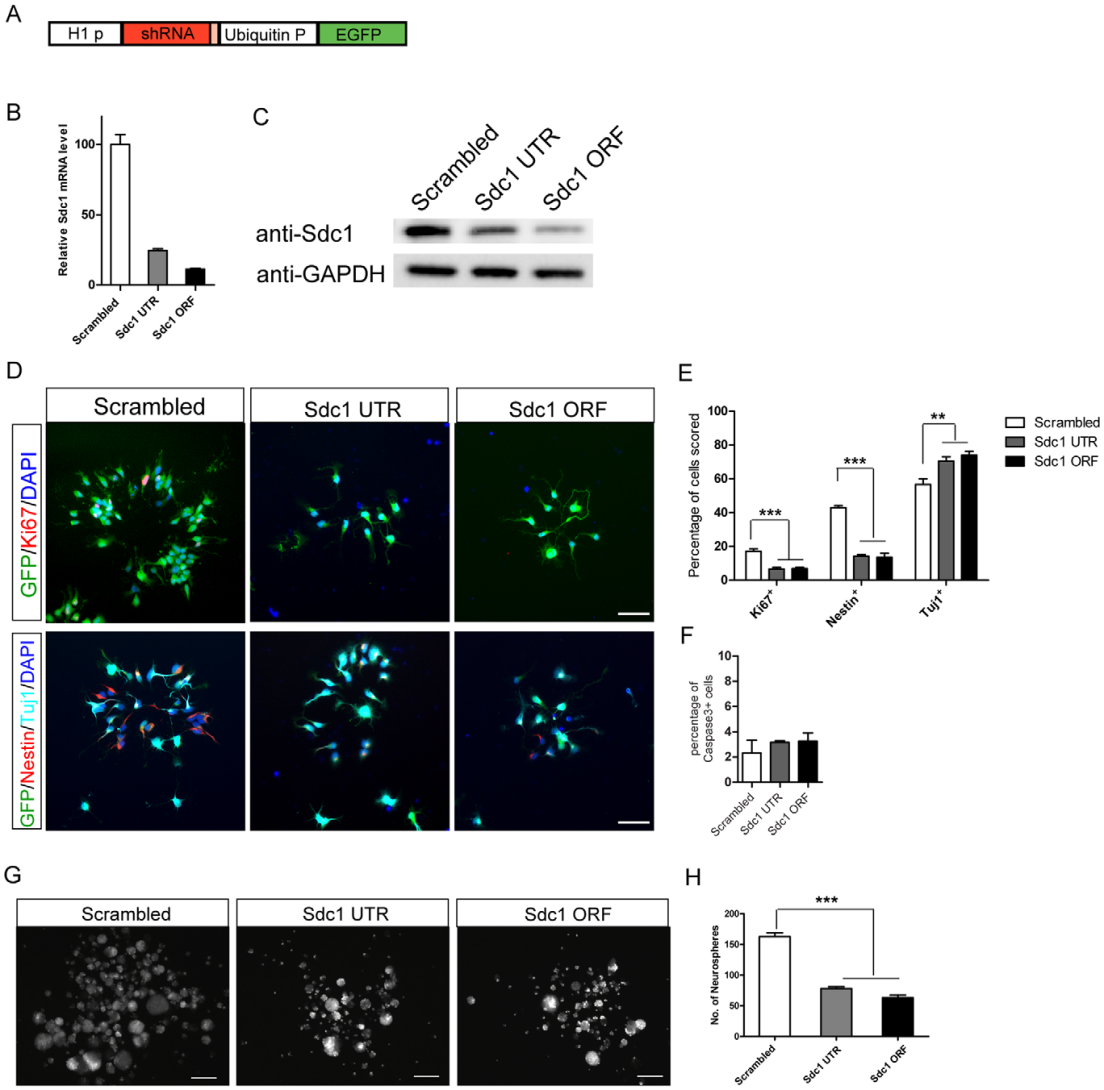
Sdc1 knockdown reduces NPC maintenance and proliferation in vitro. (A) The FUWG H1 lentiviral vector expresses a shRNA under the H1 promoter and eGFP under the ubiquitin promoter. Two different shRNAs (Sdc1 UTR and Sdc1 ORF) specifically targeting Sdc1 inhibit Sdc1 expression at mRNA (B) and protein (C) levels. (D) E11 cortical NPCs were treated with scrambled control, Sdc1 UTR and Sdc1 ORF lentiviruses and assessed at 4 DIV. Knocking down Sdc1 reduces the NPC population and their proliferation, indicated by Nestin and Ki67, respectively; knocking down Sdc1 also promotes neuronal differentiation, indicated by Tuj1 staining (scale bar = 50 um). Data are quantified in (E). (F) Knocking down Sdc1 does not increase cell apoptosis, assessed by active Caspase-3. (G) Knocking down Sdc1 reduces neurosphere generation, assessed at 7 DIV and quantified in (H) (N = 3) (scale bar = 200 um). Error bars represent S.E.M, *P<0.05, **P<0.01, ***p<0.001.

We designed two short hairpin lentiviral constructs based on the FUGW H1 vector (Fig. 2A), Sdc1 UTR and Sdc1 ORF, and showed these are capable of efficient knockdown of Sdc1. In cultured neurospheres derived from E11 cortical NPCs, these shRNA constructs reduced Sdc1 mRNA level to 25% and 11% of the scrambled control level respectively, also resulting in significant reduction in Sdc1 protein (Fig. 2B, 2C).

E11 cortical NPCs were transduced with scrambled control or Sdc1 UTR or Sdc1 ORF shRNAs and the cells were cultured at clonal density in adherent conditions. At 4 days in vitro (DIV), knocking down Sdc1 significantly reduced the progenitor population, seen by the decrease in the number of Nestin+ cells (scrambled control = 42.9%±1.3, Sdc1 UTR = 14.1%±1.1, Sdc1 ORF = 13.5%±2.4) and significantly increased neuronaldifferentiation, seen by higher levels of Tuj1+ cells (scrambled control = 56.6%±3.3, Sdc1 UTR = 74%±2.3, Sdc1 ORF = 70.5%±2.5) (Fig. 2D, 2E). The loss of NPCs could be due to reduced proliferation or increased cell death. After knockdown treatment, there was markedly decreased NPC proliferation, assessed by Ki67 labeling (scrambled control = 17.1%±1.4, Sdc1 UTR = 6.6%±1.1, Sdc1 ORF = 6.9%±0.8), but no significant change in cell apoptosis, assessed by active caspase-3 staining (Figure 2E, 2F), showing that the most likely cause of NPC depletion is reduced proliferation and increased differentiation. Consistent with this, when E11 cortical NPCs were treated with scrambled control or Sdc1 UTR or Sdc1 ORF shRNAs and grown in non-adherent conditions for 7 DIV, the Sdc1 knockdown cells generated significantly fewer neurospheres (scrambled control = 163±6, Sdc1 UTR = 78±3, Sdc1 ORF = 63±4) (Fig. 2G, 2H). These results demonstrate that Sdc1 expression is important for NPC maintenance and proliferation in vitro, and that reduced Sdc1 level leads to premature neuronal differentiation.

### Knocking down Sdc1 results in loss of NPCs and increase in neuronal differentiation in developing cortex

**Figure 3:**
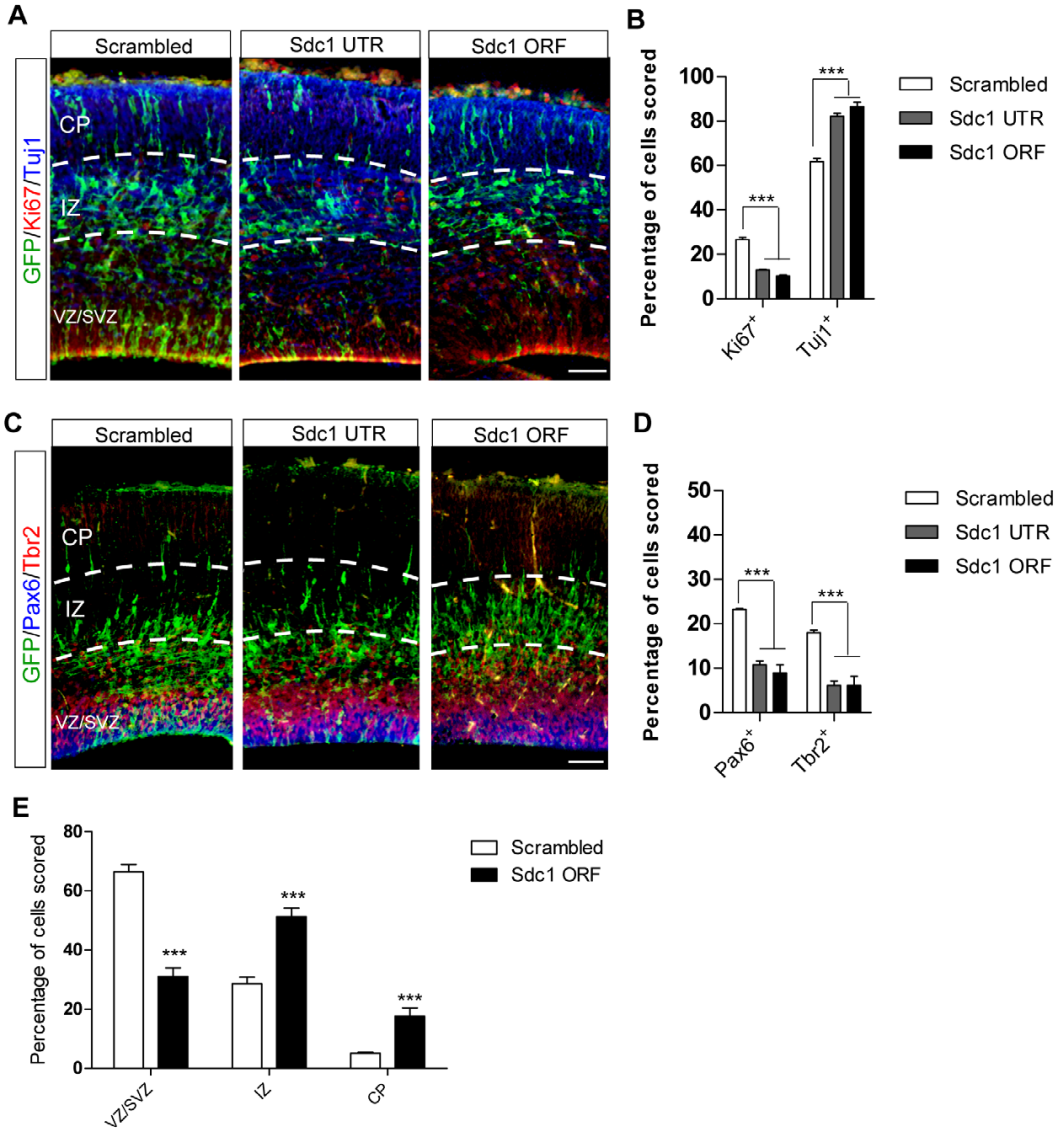
Knockdown constructs shows Sdc1 is necessary for NPC maintenance and proliferation in vivo. In utero electroporation was performed at E13 and results assessed at E15. (A) Sdc1 UTR and ORF shRNAs reduced NPC proliferation and promoted neuronal differentiation, indicated by Ki67 and Tuj1, respectively (scale bar = 50 um), as quantified in (B) (scrambled, N = 4; Sdc1 UTR, N = 3; Sdc1 ORF, N = 5). (C) Sdc1 UTR and ORF shRNAs decreased both RGC and IPC populations, indicated by reduced Pax6 and Tbr2, respectively (scale bar = 50 um) and quantified in (D) (scrambled, N = 4; Sdc1 UTR, N = 3; Sdc1 ORF, N = 5). (E) The distribution of eGFP^+^ cells in the cortical layers (scrambled, N = 5; Sdc1 ORF, N = 4). CP, cortical plate; IZ, intermediate zone; SVZ, subventricular zone; VZ, ventricular zone. Error bars represent S.E.M, *P<0.05, **P<0.01, ***p<0.001.

To examine whether knocking down Sdc1 would similarly impair NPC proliferation in vivo, we electroporated scrambled control, Sdc1 UTR or Sdc1 ORF shRNAs separately into the dorsal cortex at E13 and harvested embryos 48 hours later, at E15. Consistent with the in vitro data, knocking down Sdc1 in the developing cortex produces a decrease in NPC proliferation, assessed by Ki67 staining (scrambled control = 26.7%±1.0, Sdc1 UTR = 12.9%±0.4, Sdc1 ORF = 10.2%±0.6), and an increase in neuronal differentiation, assessed by Tuj1 staining (scrambled control = 61.8%±1.4, Sdc1 UTR = 82.1%±1.3, Sdc1 ORF = 86.5%±1.9) (Fig. 3A, 3B).

Since Sdc1 is highly expressed by both major progenitor types in the cortical germinal zones, the RGCs and the IPCs, we examined the response of these populations to Sdc1 reduction. After knocking down Sdc1 at E13 and examining the result at E15, there was a reduction in both RGCs and IPCs, assessed by Pax6 and Tbr2 staining, respectively (Pax6+: scrambled control = 23.3%±0.2, Sdc1 UTR = 10.8%±0.9, Sdc1 ORF = 8.9%±1.8; Tbr2+: scrambled control = 18.0%±0.5, Sdc1 UTR = 6.1%±1.0, Sdc1 ORF = 6.2%±2.0) (Fig. 3C, 3D). Hence Sdc1 is important for both RGC and IPC maintenance during cortical neurogenesis.

We further observed that knocking down Sdc1 resulted in a significant alteration of cell distribution between cortical zones. There were fewer cells located in the VZ/SVZ, and more cells had moved into the intermediate zone (IZ) and cortical plate (CP), consistent with increased differentiation (VZ/SVZ: scrambled control = 66.3%±2.6, Sdc1 ORF = 31.2%%±2.8; IZ: scrambled control = 28.6%±2.3, Sdc1 ORF = 51.3%%±2.9; CP: scrambled control = 5.1%±0.4, Sdc1 ORF = 17.6%%±2.9). Thus, knocking down Sdc1 in the developing cortex at mid-gestation in vivo depleted NPCs and promoted the progenitor cells to differentiate into neurons.

### Sdc1 modulates NPC responsiveness to Wnt ligand

As the major transmembrane HSPG expressed in NPCs, Sdc1 is expected to modulate the ability of NPCs to respond to key environment factors that regulate their behaviors, such as FGF and Wnt. Sdc1 has been reported to modulate Wnt signaling pathways. For example, in cultured Drosophila S2 cells, addition of the soluble Sdc1 ectodomain increased wingless (Wg) activity and led to accumulation of Armadillo/β-catenin [30]. Furthermore, Sdc1 knockout mice and are resistant to Wnt1 induced tumor formation [30], [31].

Canonical Wnt signaling pathway is a major regulator of NSC self-renewal and cortical neurogenesis [14]. In the developing cerebral cortex, Wnt plays a central role in establishment of cortical patterning [32], and blocking its downstream canonical effector β-catenin causes ventralization of progenitor cells [33]. Ectopic expression of stabilized β-catenin driven by a Nestin enhancer results in dramatic cortical progenitor cell expansion and suppression of cell cycle exit [34]. Moreover, inhibition of β-catenin in the embryonic cortex in vivo results in loss of NPCs and premature differentiation [35]. We thus decided to investigate whether Sdc1 modulates Wnt activity in cortical NPCs.

**Figure 4:**
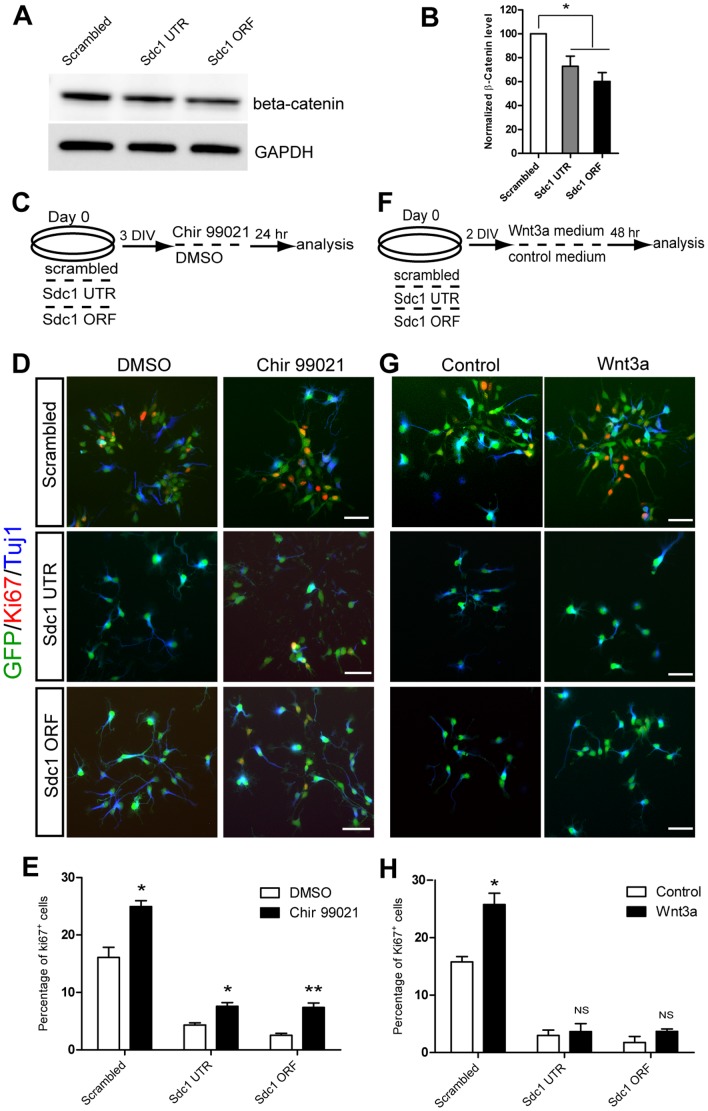
Sdc1 modulates NPC to response to Wnt ligand. (A) Knocking down Sdc1 reduced the total β-catenin protein level compared to scrambled control group, quantified in (B) after normalized to GAPDH. (C) Schematic depicting the GSK 3β inhibitor (Chir 99021) treatment experiment (D) Chir 99021 treatment partially rescued proliferation of Sdc1 UTR or Sdc1 ORF treated NPCs (scale bar = 50 um), assessed by Ki67 and quantified in (E). (F) Schematic diagram depicting the Wnt3a conditioned medium treatment experiment. (G) Wnt3a increased proliferation in scrambled control treated NPCs, but had no significant effect in Sdc1 UTR or Sdc1 ORF treated NPCs (scale bar = 50 um), assessed by Ki67 and quantified in (H). Error bars represent S.E.M, *P<0.05, **P<0.01, ***p<0.001, NS = not significant.

To examine this directly, we assessed the level of β-catenin in cortical cells after Sdc1 knockdown, and found a significant decrease compared to scrambled control (Fig. 4A, 4B), suggesting that loss of Sdc1 expression in NPCs negatively regulates β-catenin stability and accumulation. To further examine if this is due to impairment of the Wnt/β-catenin signaling pathway, we asked whether Sdc1 knockdown could be circumvented by addition of downstream Wnt activators. Hence we treated cultured NPCs transduced with scrambled or Sdc1 ORF and UTR shRNA with Chir 99021 (500 nM), a potent inhibitor of GSK3β, or DMSO (vehicle control), provided at 3 DIV for 24 hours, then fixed the cells and examined NPC proliferation, assessed by Ki67 staining (Fig. 4C). We found that treatment with Chir 99021 increased NPC proliferation in the scrambled control group (DMSO = 16.1%±1.8; Chir 99021 = 24.9%±1.0) and that the reduction of NPC proliferation following Sdc1 knockdown was partially rescued after Chir 99021 treatment (Sdc1 UTR: DMSO = 4.3%±0.4, Chir 99021 = 7.6%±0.7; Sdc1 ORF: DMSO = 2.5%±0.4, Chir 99021 = 7.4%±0.8) (Fig. 4D, 4E). Hence reducing Sdc1 in NPCs impairs the activity of the canonical Wnt signaling pathway.

If Sdc1 is modulating Wnt/β-catenin activity through concentrating and stabilizing Wnt proteins at the cell surface, Sdc1 knockdown should prevent the NPCs from responding to exogenous Wnt protein. To test this, we treated cultured NPCs transduced with scrambled or Sdc1 UTR and ORF shRNA with Wnt3a conditioned medium (2 ng/ml) at 2 DIV for 48 hours, then fixed the cells for analysis at 4 DIV (Fig. 4F). We found that treatment with Wnt3a significantly increased NPC proliferation in the scrambled control group, assessed by Ki67 staining (control = 15.8%±0.9; Wnt3a = 25.8%±2.0), but Sdc1 UTR and ORF shRNA treatments prevented the cells from responding to added Wnt3a (Sdc1 UTR: control = 3.0%%±0.9, Wnt3a = 3.7%±1.4; Sdc1 ORF: control = 1.6%±1.0, Wnt3a = 3.7%±0.4) (Fig. 4G, 4H). These results indicate that loss of Sdc1 negatively regulates NPC proliferation by modulating the NPC responsiveness to Wnt ligand.

## Discussion

In this study we investigated the expression and function of the major membrane associated HSPG Sdc1 in the developing cerebral cortex. We show that Sdc1 is highly expressed in cortical germinal zones in both RGCs and IPCs. Loss of Sdc1 results in loss of the progenitor populations and abnormally increased neurogenesis, seen both in clonal studies in vitro and in the developing cortex in vivo. We found that Sdc1 is required for Wnt stimulated NPC proliferation. Hence we identify Sdc1 as a key niche factor during cortical neurogenesis, and an important modulator of canonical Wnt signaling in cortical progenitor populations.

Studies in Drosophila have demonstrated that Wingless (Wg) signaling is critically dependent upon interactions with HSPGs which chaperone the hydrophobic Wg molecule to enable distribution into signaling gradients [36]. Indeed, mutations in HSPG synthesis phenocopy Wg loss [36]. Although prior work has shown that Wnt signaling is important for the normal balance of cell division and differentaition in the cortex, the specific HSPGs that aid cortical Wnt signaling have not been elucidated. Here we demonstrate that Sdc1 facilitates Wnt signaling in NPCs during cortical neurogenesis. Consistent with this, we note that the knockdown of Sdc1 in vivo phenocopies the inhibition of β-catenin signaling already reported in the cortex, causing progenitor cells to prematurely exit the cell cycle, differentiate into neurons, and migrate to the cortical plate [35]. It will be fascinating to understand in the future how Sdc1 facilitates Wnt signaling, perhaps by affecting the effective concentration at the NPC surface, presenting a multivalent growth factor environment, or preventing Wnt from aggregating in the aqueous environment, as found from isolated cell studies [37].

In addition to the Wnt signaling pathway, Sdc1 is involved in other signaling pathways in a variety of cell types under different physiological and pathological conditions [38], [39], [40], [41]. For example it can alter FGF signaling and we know FGF is a mitogen for cortical NPCs, acting in a concentration-dependent manner [42]. Interestingly we were unable to see a marked reduction in the FGF pathway effector phospho-p44/42 MAPK (ERK1/2) after Sdc1 knockdown (not shown) so it may be less important as a modulator of this pathway compared to the canonical Wnt pathway. However, alternative FGF-dependent signaling pathways such as the PI3K/AKT pathway that can contribute to the proliferation of neural progenitor cells [43], remain to be investigated in this system. Indeed, given that a GSK3 beta inhibitor causes only partial rescue of Sdc1 knockdown, Sdc1 could modulate other environmental regulators. For example, one candidate protein that could interact with Sdc1 is CASK, a member of the membrane-associated guanylate kinase homolog (MAGUK) superfamily that is known to interact with syndecan-3 in other systems [20]. Like Sdc1 knockout mice, CASK mutant mice are smaller than normal [31], [44]. In human patients, mutations of CASK cause microcephaly [45]. In the developing cortex, CASK is expressed in NPCs and migrating neurons [20], [46] and it will be worth investigating whether Sdc1 interacts with CASK in NPCs, affording another way to regulate cortical neurogenesis. Furthermore, Sdc1 is also found in the nucleus, suggesting a role in transcriptional regulation [47], [48], which is another point for future studies.

Sdc1 is enriched in NPCs but is not expressed at detectable levels by neurons in the developing cerebral cortex. As cortical neurogenesis proceeds, the expression level of Sdc1 is reduced [27]. In the adult rat, Sdc1 is not detected in the forebrain, suggesting that it plays a specific role during cortical development in regulating NPC maintenance and proliferation [20]. We found that the effect of knocking down Sdc1 in vitro at E11 and in vivo at E13 was similar – a promotion of neuronal differentiation, however it is possible that at different stages of development, Sdc1 plays a different role. Sdc1 has been associated with tumor progression in a number of different tumor cell types [30], [40]. In malignant gliomas, Sdc1 is abnormally up-regulated [49]. Wnt signaling is critical for malignant glioma progression [50]. It will be worthwhile to explore whether Sdc1 is involved in gliomagenesis through facilitating the Wnt signaling pathway, as we have found for normal NPCs, as this could provide an extracellular target for combating tumor growth.
