# Correction: The Niche Factor Syndecan-1 Regulates the Maintenance and Proliferation of Neural Progenitor Cells during Mammalian Cortical Development

**DOI:** 10.1371/annotation/76cf3bd3-f842-489f-8ad2-4c016447b84c

**Published:** 2012-11-16

**Authors:** Qingjie Wang, Landi Yang, Caroline Alexander, Sally Temple

In the first sentence of the section "Plasmid and Lentivirus Preparation" of the Materials and Methods, some of the sequences are incorrect. It should read: shRNA plasmids were generated by inserting the hairpin oligonucleotides (Scrambled control: TTCTCCGAACGTGTCACGT; Sdc1 ORF: CCACCAAGCAGGAGGAGTT; Sdc1 UTR: CCACACCTGTCGTCCACTC) into the FUGW-H1 lentiviral construct as previously described [28].

